# Non-adherence to Anti-diabetic Prescriptions Among Type 2 Diabetes Mellitus Patients in the Kurdistan Region of Iraq

**DOI:** 10.7759/cureus.60572

**Published:** 2024-05-18

**Authors:** Soran Hamalaw, Aso Hama Salih, Sardar Weli

**Affiliations:** 1 Nursing Department, Sulaimani Polytechnic University, Sulaymaniyah, IRQ

**Keywords:** type 2 diabetes mellitus, iraq, kurdistan, non-adherence, medication adherence

## Abstract

Background: Treatment adherence is a primary key in controlling diabetes disease. The study aims to determine the prevalence of treatment adherence in type 2 diabetes mellitus (T2DM) patients, investigate the potential influence of adherence on elevated blood glucose levels, and identify the key factors which play a role in non-adherence to the prescribed drugs.

Method: A cross-sectional study method was utilized to collect data from all T2DM patients at the Diabetic and Endocrine Centre and Shar Hospital in Sulaymaniyah city in the Kurdistan region of Iraq from February 2022 to April 2022. The data collection was performed through a structured questionnaire. The prevalence of drug adherence was assessed using the Morisky Medication-Taking Adherence Scale (4-item), and the glycated hemoglobin test (A1C) was used to determine the blood glucose level.

Result: A total of 300 participants were studied, and more than half of them (192; 64%) revealed that they did not adhere to their anti-diabetic medications. Non-adherence was significantly associated with higher A1C. Several barriers to non-adherence were identified as multiple medications, feeling the dose given is high, lack of finance, and side effects by 209 (70%), 116 (39%), 113 (38%), and 103 (34%), respectively.

Conclusion: The current study's result revealed that most T2DM patients have no adherence to their medication. This non-adherence is significantly linked to higher A1C levels, emphasizing the critical role of medication compliance in managing diabetes effectively. The study also sheds light on the multiple barriers such as taking multiple prescriptions, the perception that the dose is excessive, lack of finances, and experiencing side effects, which contribute to non-adherence among diabetes patients. These findings underscore the need for healthcare providers to address these barriers and develop tailored strategies to enhance medication adherence among individuals with diabetes.

## Introduction

Type 2 diabetes mellitus (T2DM) is one of the chronic illnesses that, in 2021, 537 million adults, in other words, more than a tenth of the planet's population, were estimated to be suffering from [1,2]. This number is predicted to rise to 643 million by 2030. Over three-quarters of adults with T2DM dwell in low- and middle-income countries [2]. Diabetes is regarded as the leading cause of disturbing the life aspects of the individual. The disease is depicted by elevated blood glucose levels, which can result in many serious health complications, including heart disease, stroke, blindness, and kidney failure [3].

Treatment adherence is a vital component of managing diabetes disease, which involves a complex treatment regimen and lifestyle changes. However, many patients with T2DM do not adhere to their medication regimen. This may result in inadequate glucose control and a higher chance of complications. The prevalence of poor glycemic control (A1C >7%) among adults is estimated at 61.92% worldwide [4]. Treatment adherence failure is an essential problem impacting the patient and the healthcare system. Treatment adherence is the primary key to managing diabetes disease, which involves a complex treatment regimen and lifestyle changes.

Background

Diabetes patients, like other chronic diseases, have poor adherence to treatment. When it comes to poor treatment adherence, diabetes is the second most chronic condition out of 17. Patients who were not following their prescribed regimen experienced increased hospitalization and mortality rates across all causes [5]. The prevalence of non-adherence to diabetes prescriptions ranges from 25% to 75%, depending on the country. For instance, a study in Pakistan found that 62% of T2DM patients did not take their anti-diabetic medicine as directed [6]; similar findings were reported in Cameroon (54.4%) [7], Malaysia (39.6%) [8], Ethiopia (42%) [9], and Switzerland (60%) [10].

Many factors can impact the adherence rate, including patient-related factors such as age, sex, education, income, health literacy, comorbidities, and mental health status; medication-associated factors such as the complexity of the therapeutic regimen, the number of medications, the side effects of the medicines, and the cost of the drugs; healthcare system-associated factors such as the availability of healthcare providers, the quality of care, and the cost of healthcare; and psychosocial factors such as stress, anxiety, depression, and social support. These facts suggest that each society may have different adherence prevalence and needs a specific assessment to determine the factors which affect the adherence rate.

Objective

The current study is designed to determine the prevalence of adherence to anti-diabetic medications in T2DM patients and the role of medication adherence on poor glycemic control status. The study also aimed to identify the factors that lead to non-adherence to medicine in people with T2DM in the Kurdistan region of Iraq.

## Materials and methods

Study design

A cross-sectional study design was used to conduct the study. The data was collected from patients who visited the Diabetic and Endocrine Centre and Shar Hospital in Sulaymaniyah city in the Kurdistan region of Iraq from February 2022 to April 2022.

Inclusion criteria

All adult patients with T2DM and on anti-diabetic medications in the mentioned hospitals were eligible to participate in the current study. Non-diabetes patients and type 1 diabetes patients were excluded.

Data collection

A structured questionnaire containing 35 questions was used for data collection (see Appendices). It covers various demographic factors, medical history, medication adherence behaviors, attitudes toward medication, barriers to adherence, and factors influencing medication use. The Morisky Medication-Taking Adherence Scale (MMAS) (4-item) [11] was utilized to assess the prevalence of medication adherence, while to determine blood glucose level, the A1C was measured. The questionnaire was administered via a face-to-face interview with the participants.

Data analysis

The collected data was screened, coded, and added to the Microsoft Excel sheet. Then, the Excel sheet was imported to IBM SPSS Statistics for Windows, Version 26.0 (Released 2019; IBM Corp., Armonk, New York, United States) for analysis. The scores were computed (Yes=0 and No=1), and the patient's total score was obtained using the following cut-offs to interpret the patient's adherence: score of 0 or 1=poor adherence; score of 2 or 3=moderate adherence; and score of 4=good adherence. In the current study, participants were categorized into two groups, those considered adhesive (with a total score of 4 on MMAS-4) and those classified as non-adherent (with a total score of 3 or lower on MMAS-4), using a three-point threshold as the criterion. Descriptive statistics were applied to show the demographic characteristics of respondents and other different factors. The relationship between variables and non-adherence was analyzed by cross-tabulation and using the Fisher exact test and chi-squared test for categorical variables. The independent t-test and ANOVA test were applied to evaluate the mean difference between the groups. A p-value of less than 0.05 was taken as a statistically significant cut point.

Ethical considerations

Ethical approval was obtained from the Health Research Ethics Committee (the institutional review board (IRB)) of the College of Health and Medical Technologies, Sulaimani Polytechnic University, under project number CH00027, on November 08, 2021. Before administering the questionnaire, the purpose of the study was disclosed to all respondents. They were assured that anonymity, confidentiality, and privacy would be secured. They were also informed that participation in this study is voluntary and they are free to withdraw whenever they want.

## Results

Characteristics of the sample

A total of 300 T2DM patients 57.8±9.95 years old on average, 181 (60.3%) females and 119 (39.7%) males, participated in the study. The majority of the participants were non-smokers (269; 89.7%) and non-alcoholics (296; 98.7%). Two hundred and sixty-eight (89.3%) were married, 127 (42.3%) were with high income, and 108 (36%) were classified as middle income. The mean A1C of the patients was 8.92%, with a standard deviation of 2.2. The characteristics of the study participants are presented in Table 1.

**Table 1:** Characteristics of the study participants

Characteristics		Frequency N	Percentage %
				Age in years	Mean (standard deviation)	57.76	9.95
Gender	Male	119	39.7
	Female	181	60.3
Smoking status	Yes	31	10.3
	No	269	89.7
Alcoholic consumption	Yes	4	1.3
	No	296	98.7
Residency	Inside Sulaymaniyah city	253	84.3
	Outside Sulaymaniyah city	47	15.7
Marital status	Unmarried	7	2.3
	Married	268	89.3
	Divorced	3	1
	Widow or widower	22	7.3
Income	High income	127	42.3
	Middle income	108	36
	Low income	65	21.7
HBA1C	Mean (standard deviation)	8.92	2.2

Results

**Figure 1:**
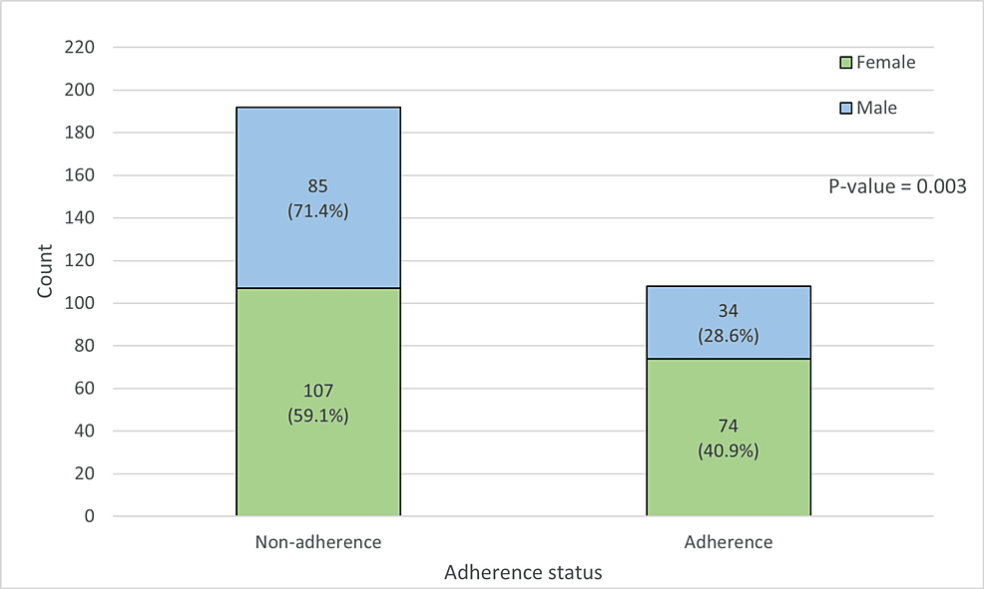
The male-female medication adherence among the participants

The prevalence of medication non-adherence was 192 (64%). There was a significant difference between genders regarding adherence to T2DM medications. Non-adherence was more prevalent among males (85; 71.4%) compared to females (107; 59.1%), with a p-value of 0.03 (Figure 1). The individuals' mean age did not significantly differ according to adherence status. Non-adherence was significantly higher in patients who live outside the city (38; 80.9%) than those who live inside the city (154; 60.9%), with a p-value of 0.009.

The findings showed a significant association between adherence status and the A1C level of the patients; compared to the adherence group, non-adherence participants had a greater likelihood of having a higher A1C (p=0.009).

A one-way ANOVA was applied to assess the effect of medication adherence classes on A1C measurement in T2DM patients. The mean A1C was 9.28 for poor adherent (high), 9.15 for moderate adherent (moderate), and 8.47 for good adherent (low). A statistically significant difference was found between the adherence to diabetic medications on A1C (F (3,453)=32.871; p=0.033).

Regarding the MMAS questions, 174 (58%) admit forgetting to take their medications frequently. About 123 (41%) of the participants disclosed that they were careless regarding taking their medications. Forty-one patients (13.7%) confirmed that they would stop taking the drug when they felt better, and 25 (8.3%) discontinued the medications when feeling worse (Table 2).

**Table 2:** MMAS (4-item) MMAS: Morisky Medication-Taking Adherence Scale

#	Items	Yes, n (%)	No, n (%)
1	Do you ever forget to take your diabetic medication?	174 (58%)	126 (42%)
2	Are you careless at times about taking your medicine?	123 (41%)	177 (59%)
3	When you feel better, do you sometimes stop taking your diabetic medication?	41 (13.7%)	259 (86.3%)
4	If you feel worse when you take your diabetic medication, do you stop taking it?	25 (8.3%)	275 (91.7%)

Several barriers were identified for no adherence to T2DM drugs as prescribed by the physician. Use of multiple medications for other diseases, feeling of the high dose prescribed, lack of finances, and side effects were the most common barriers to non-adherence by 209 (69.7%), 116 (38.7%), 113 (37.7%), and 103 (34.3%), respectively (Table 3).

**Table 3:** Barriers to non-adherence to anti-diabetic medications

Items	Frequency N=300	Percentage %
Multiple medications? Use of other drugs	209	69.7
Feeling the dose given is high?	116	38.7
Lack of finance?	113	37.7
Side effects?	103	34.3
The lack of knowledge about prescribed anti-diabetic medications	90	30
Interferes with my meal plan?	82	27.3
Feeling drugs are not effective?	69	23
The physician did not give information on anti-diabetic medications	42	14
Poor family support?	34	11.3

The intentional adherence of the patients was assessed using several questions about behavior and beliefs. Table 4 shows the frequency and percentage of respondents who answered the questions with "Yes." The patients who admitted they had modifications in the dose and timing of their prescribed medication had a higher probability of being in the non-adherent group, with a p-value of 0.042 and <001, respectively. In response to the questions "Do you think anti-diabetic medications are essential to control your blood glucose?", "Were you involved in treatment decisions?", and "Do you feel comfortable asking questions to your doctor?", no significant statistical association between the responses to these questions and adherence to anti-diabetic prescriptions was observed.

**Table 4:** Association of behaviors and beliefs of the patients and adherence status to T2DM medications T2DM: type 2 diabetes mellitus

Items	Total frequency	Non-adherence	Adherence	P-value
	N=300 (%)	n (%)	n (%)	
Do you make your own modifications in the dose of drugs prescribed?	76 (25.3)	56 (29.2)	20 (18.5)	0.042
Do you make your own modifications in the timing of anti-diabetic drugs?	131 (43.7)	102 (53.1)	29 (26.9)	<0.001
Do you think anti-diabetic medications are important to control your blood sugar?	110 (36.7)	76 (39.6)	34 (31.5)	0.162
Were you involved in treatment decisions?	246 (82)	156 (81.3)	90 (83.3)	0.652
Do you feel comfortable asking questions to your doctor?	275 (91.7)	174 (90.6)	101 (93.5)	0.384

## Discussion

Medication therapy is the basis of diabetes disease management. Nevertheless, most patients in the current study were unsuccessful in adhering to their anti-diabetic treatment. Lack of adherence to drug therapy is linked to inadequate glycemic control and diabetic complications [6], leading to the massive cost of healthcare as well [12]. Medication therapy is the basis of diabetes disease management. Nevertheless, most patients were unsuccessful in adhering to their anti-diabetic treatment.

In this study, 64% of participants did not take their anti-diabetic medications as prescribed. This finding highlights the magnitude of the problem in this patient population and underscores the need for interventions to improve medication adherence. A similar result was obtained in Cameroon (54.4%) [7], Ethiopia (41.5%) [13], another study (54.1%) [14], and Malaysia (65%) [15]. A study in Northwest Ethiopia presented two-thirds (76.9%) of their contributors were non-adherent to their anti-diabetic medication, which is slightly higher than this study result [16]. However, significantly lower levels of non-adherence have been reported in Spain, Nigeria, and Uganda, recording levels of 31.68%, 27.5%, and 16.7%, respectively [17-19]. The dissimilarities in healthcare services, socioeconomic conditions, and utilized metric tools for adherence evaluation across the study settings could be related to the adherence rate variances.

An interesting observation was the substantial association between gender and treatment adherence. Non-adherence was more prevalent among females (60.3%) than males (39.7%). This gender disparity in adherence is consistent with previous research that showed that women showed higher degrees of diabetes distress and depression along with lower levels of medication adherence [20]. There are a few possible explanations for this disparity. One possibility is that women may be more likely to experience side effects from T2DM medications. The first-line treatments for T2DM in the region are usually metformin and sulfonylureas. The most common side effects of metformin include diarrhea, nausea, vomiting, abdominal discomfort, bloating, weakness, muscle pain, trouble breathing, unusual sleepiness, stomach discomfort, dizziness, and slow or irregular heartbeat. Sulfonylureas include glyburide, glipizide, and glimepiride. The common side effects of sulfonylureas include hypoglycemia, weight gain, and gastrointestinal upset. Experiencing side effects can make the patient less likely to take these drugs consistently.

Additionally, women may face more barriers to accessing care, such as childcare or transportation challenges. Finally, social and cultural factors may also play a role, such as the expectation that women will take care of others' needs before their own. This variance may warrant further investigation into the underlying reasons for this difference. Contrary to some expectations, there was no significant difference in medication adherence based on the mean age of the participants. This result suggests that age alone may not be a substantial determinant of medication adherence among T2DM patients in this population.

Another reason for non-adherence to anti-diabetic medicines is literacy and attending higher education because the prevalence was much higher in rural areas, where most of their residents were illiterate people [21,22]. A study defined a lack of knowledge and awareness of diabetes as contributing to drug adherence [23].

One of the key findings was the significant association between medication adherence and A1C levels. Non-adherent patients were likelier to have higher A1C levels, indicating poorer glycemic control. A similar result was reported in a study in Spain [17]. This underscores the critical importance of medication adherence in achieving and maintaining target blood glucose levels in T2DM patients.

The study identified several common barriers to medication adherence, including using multiple drugs for other diseases, the perception of a high medication dose, financial constraints, and concerns about side effects [24]. In comparison, family support was at the bottom of the drug adherence barriers. The patients who had low finances and could not purchase prescribed drugs were also found to not adhere to medication [25]. These barriers align with previous research and highlight the multifaceted nature of non-adherence.

Several researchers supported this study's result that comorbidity, low income, poor information about the diabetic disease, and medication educational status of the patients were the critical factors of the drug adherence problem, especially in low-income nations [14,26]. Poor control of blood glucose due to low levels of drug adherence may increase the negative health impacts and medical finance, and it has severely affected the healthcare system's maintainability [27].

The assessment of intentional adherence revealed that patients who reported making modifications in the dose and timing of their prescribed medication were more likely to be in the non-adherent group. This behavior emphasizes the importance of patient self-management demeanors in medication adherence. Additionally, certain beliefs and attitudes about medication, such as its importance in controlling blood glucose, involvement in treatment decisions, and comfort in asking questions to the doctor, were not statistically associated with adherence. These feelings suggest that patient education and communication with healthcare providers may not be the sole determinants of adherence and that other factors play a significant role.

Strengths and limitations of the work

The study provides crucial information about medication adherence in this region; however, it includes only patients who visited specific hospitals in Sulaymaniyah city, which might not represent the entire population of T2DM patients in the region. This could introduce selection bias. Additionally, the study evaluated medication adherence using self-reported data, which is prone to social desirability and recall bias. Electronic monitoring or pill counts are examples of objective measurements of adherence that could improve this aspect of the study. Finally, the findings may not be directly applicable to T2DM populations in other regions or countries, as factors influencing adherence can vary significantly.

Recommendations for further research

Further research in this area should prioritize longitudinal studies to establish causal connections between factors and non-adherence to treatment among T2DM patients. It is crucial to incorporate objective measures of medication adherence, such as electronic monitoring, to validate self-reported data. Expanding the research to include diverse healthcare settings and populations will enhance the generalizability of findings. Additionally, intervention studies should be conducted to evaluate the effectiveness of targeted interventions in addressing the identified barriers to adherence. These steps will contribute to a more comprehensive understanding of medication adherence in T2DM and inform the development of effective strategies for improving patient outcomes.

Implications for policy and practice

The study's findings have significant implications for both policy and practice in diabetes management. Policymakers should consider the development and implementation of patient education programs aimed at raising awareness about the importance of medication adherence in T2DM management, alongside addressing systemic factors like healthcare accessibility and affordability. In clinical practice, healthcare providers should prioritize regular monitoring of A1C levels in T2DM patients, particularly those with identified barriers to adherence, and tailor interventions to address specific issues such as multiple medications, high doses, and side effects. These proactive measures can contribute to better glycemic control and overall improved health outcomes among T2DM patients.

## Conclusions

The current study provides evidence that the prevalence of non-adherence to anti-diabetic medications is high in people with diabetes. Additionally, non-adherence was associated with higher A1C levels. This means that people who do not adhere to their drugs are more likely to have poor blood glucose control, which may increase the risk of complications. The barriers to adherence included multiple medications, feeling the dose is too high, lack of finances, and side effects. Interventions to improve medication adherence are needed to help people with diabetes manage their condition and prevent complications.

## Appendices

Adherence to type 2 diabetes mellitus (T2DM) medication questionnaire

Questionnaire ID: ____________________                                               Interviewer: ____________________

Thank you for participating in our study focused on understanding medication adherence among individuals with diabetes. Your input is crucial in improving diabetes management and enhancing overall health outcomes.

Purpose of the Questionnaire

This questionnaire aims to assess your adherence to diabetic medications. By sharing your experiences, you contribute valuable insights that can inform healthcare practices and support better patient outcomes.

Confidentiality

Again your participation is voluntary, and your responses will remain confidential. We do not collect any personally identifiable information. Please answer each question honestly and to the best of your ability.

**Table 5:** Non-adherence assessment-related questions T2DM: type 2 diabetes mellitus

Question	Possible responses	Check
1. Age	__________ years	
2. Gender	Male	
	Female	
3. Level of education	Illiterate	
	Reads and writes	
	Primary school graduate	
	Preparatory school graduate	
	College and institute graduate	
4. Residency area	Inside cities	
	Outside cities	
5. Marital status	Single	
	Married	
	Divorced	
	Widow or widower?	
6. Number of children	__________	
7. Occupation	Government employed	
	Private employed	
	Retired	
	Housewife	
	Jobless	
	Others: __________	
8. Monthly income	Sufficient	
	Barely sufficient	
	Insufficient	
9. Smoking	Yes	
	No	
10. Drink alcohol	Yes	
	No	
11. Duration of T2DM	<1 year	
	1-3 years	
	4-8 years	
	9-12 years	
	>13 years	
12. Treatment	Insulin therapy	
	Oral drug therapy	
	Combination therapy	
13. Last HbA1C test (see test result)	__________ mmol/L	
14. Do you ever forget to take your diabetic medication?	Yes	
	No	
15. Are you careless at times about taking your diabetic medication?	Yes	
	No	
16. When you feel better, do you sometimes stop taking your diabetic medication?	Yes	
	No	
17. If you feel worse when you take your diabetic medication, do you stop taking it?	Yes	
	No	
18. Do you take anti-diabetic drugs as advised by your doctor?	Yes	
	No	
19. Do you make your own modifications in the dose of drugs prescribed?	Yes	
	No	
20. Do you make your own modifications in the timing of anti-diabetic drugs?	Yes	
	No	
21. Do you know the importance of anti-diabetic medication?	Yes	
	No	
22. Were you involved in treatment decisions?	Yes	
	No	
23. Do you feel comfortable asking questions to your doctor?	Yes	
	No	
24. Lack of finance?	Yes	
	No	
25. Feeling the drug is not effective?	Yes	
	No	
26. Interferes with my meal plan	Yes	
	No	
27. Side effects?	Yes	
	No	
28. Multiple medications?	Yes	
	No	
29. Feeling the dose given is high?	Yes	
	No	
30. Did your physician give information on anti-diabetic medications?	Yes	
	No	
31. Do you have good knowledge about anti-diabetic medications prescribed to you?	Yes	
	No	
32. Do you regularly monitor your blood glucose?	Yes	
	No	
33. Do your family members remind you or assist you in taking your medications as prescribed?	Yes	
	No	
34. Are there other things affecting your regular use of T2DM medication?	Yes	
	No	
35. If yes, please specify	__________	

Thank you!
